# Population genetic analyses inferred a limited genetic diversity across the *pvama-1* DI domain among *Plasmodium vivax* isolates from Khyber Pakhtunkhwa regions of Pakistan

**DOI:** 10.1186/s12879-022-07798-1

**Published:** 2022-10-30

**Authors:** Ibrar Ullah, Sahib Gul Afridi, Muhammad Israr, Hizbullah Khan, Sulaiman Shams, Komal Zaib, Huong Giang Le, Jung-Mi Kang, Byoung-Kuk Na, Asifullah Khan

**Affiliations:** 1 Department of Biochemistry, Abdul Wali Khan University Mardan (AWKUM), Mardan, 23200 Pakistan; 2 Department of Forensic Sciences, University of Swat, Swat, 19200 Pakistan; 3 CAS Key Laboratory of Molecular Virology and Immunology, The Center for Microbes, Development and Health, Institut Pasteur of Shanghai, Chinese Academy of Sciences, Shanghai, China; 4 University of Chinese Academy of Sciences, Beijing, China; 5 Department of Parasitology and Tropical Medicine, Department of Convergence Medical Science, Institute of Health Sciences, Gyeongsang National University College of Medicine, Jinju, 52727 Korea

**Keywords:** *Plasmodium vivax*, Apical membrane antigen-1, Genetic diversity, Khyber Pakhtunkhwa, Pakistan

## Abstract

**Background:**

*Plasmodium vivax* apical membrane antigen-1 (*pvama-1*) is an important vaccine candidate against Malaria. The genetic composition assessment of *pvama-1* from wide-range geography is vital to plan the antigen based vaccine designing against Malaria.

**Methods:**

The blood samples were collected from 84 *P. vivax* positive malaria patients from different districts of Khyber Pakhtunkhwa (KP) province of Pakistan. The highly polymorphic and immunogenic domain-I (DI) region of *pvama-1* was PCR amplified and DNA sequenced. The QC based sequences raw data filtration was done using DNASTAR package. The downstream population genetic analyses were performed using MEGA4, DnaSP, Arlequin v3.5 and Network.5 resources.

**Results:**

The analyses unveiled total 57 haplotypes of *pvama-1* (DI) in KP samples with majorly prevalent H-14 and H-5 haplotypes. Pairwise comparative population genetics analyses identified limited to moderate genetic distinctions among the samples collected from different districts of KP, Pakistan. In context of worldwide available data, the KP samples depicted major genetic differentiation against the Korean samples with *Fst* = 0.40915 (P-value = 0.0001), while least distinction was observed against Indian and Iranian samples. The statistically significant negative values of Fu and Li’s D* and F* tests indicate the evidence of population expansion and directional positive selection signature. The slow LD decay across the nucleotide distance in KP isolates indicates low nucleotide diversity. In context of reference *pvama-1* sequence, the KP samples were identified to have 09 novel non-synonymous single nucleotide polymorphisms (nsSNPs), including several trimorphic and tetramorphic substitutions. Few of these nsSNPs are mapped within the B-cell predicted epitopic motifs of the *pvama-1*, and possibly modulate the immune response mechanism.

**Conclusion:**

Low genetic differentiation was observed across the *pvama-1* DI among the *P. vivax* isolates acquired from widespread regions of KP province of Pakistan. The information may implicate in future vaccine designing strategies based on antigenic features of *pvama-1.*

**Supplementary Information:**

The online version contains supplementary material available at 10.1186/s12879-022-07798-1.

## Background

Malaria is an acute febrile infectious disease caused by vector-borne apicomplexan parasites of the genus *Plasmodium*. The *P. vivax* and *P. falciparum* are predominant species responsible for malaria [1]. The *P. vivax* is most widely distributed human malaria parasite, endemic in tropical and subtropical countries of Asia, South Pacific, Central and South America, Middle East, and North Africa [2]. According to the latest WHO report, about 229 million cases and 40,900 deaths occur due to malaria in 2019 [3].

Treatment and control of malaria have become a serious challenge due to drug resistance and lack of effective vaccines. The wide-range distribution, antigenic variation, relapsing and co-infection led to a collective interest towards the development of effective vaccine against *P. vivax* [4]. The implementation of RTS,s/AS01 vaccination was started in three African countries during 2019 and considered effective against malaria to date. Furthermore, the R21/Matrixs-M vaccine was tested among children in Bukina Faso and reported to meet the WHO’s goal up to 77% against malaria [5]. Several antigens of *Plasmodium* species such as apical membrane antigen-1 (AMA-1), Circumsporozoite proteins (CSP), Merozoite surface proteins (MSP) and Duffy binding protein (DBP) are reported as potent malarial vaccine candidates’ targets [6].

The genetic composition assessment of vaccine candidates’ loci is indispensable in modern-age to plan an effective vaccination strategy. There are ample of studies suggesting the AMA-1 of *Plasmodium* species as promising malaria vaccine candidate antigens [7]. The AMA-1 is a type-I integral membrane protein with molecular size of 83 kDa, mainly expressed in the merozoite and sporozoite stages of *Plasmodium* parasites [8, 9]. The main biological function of AMA-1 is not well known so far, however the stage-specific expression and localization suggest its crucial role during invasion of erythrocytes and hepatocytes by malarial parasites [10–12]. The protein consists of cysteine rich ectodomain having three separated domains (i.e. Domain I, II, and III), a conserved cytoplasmic region and a transmembrane domain [13]. The ectodomain of the protein is highly immunogenic and evokes natural immune responses among patients infected by *P. falciparum* and *P. vivax* [14–17]. Furthermore, the protein AMA-1 reported to elicit the antibody production that effectively halt the invasion of erythrocytes by malarial parasite and confers protective immune responses [18]. This suggesting the AMA-1 as a leading malarial vaccine candidate.

**Fig. 1:**
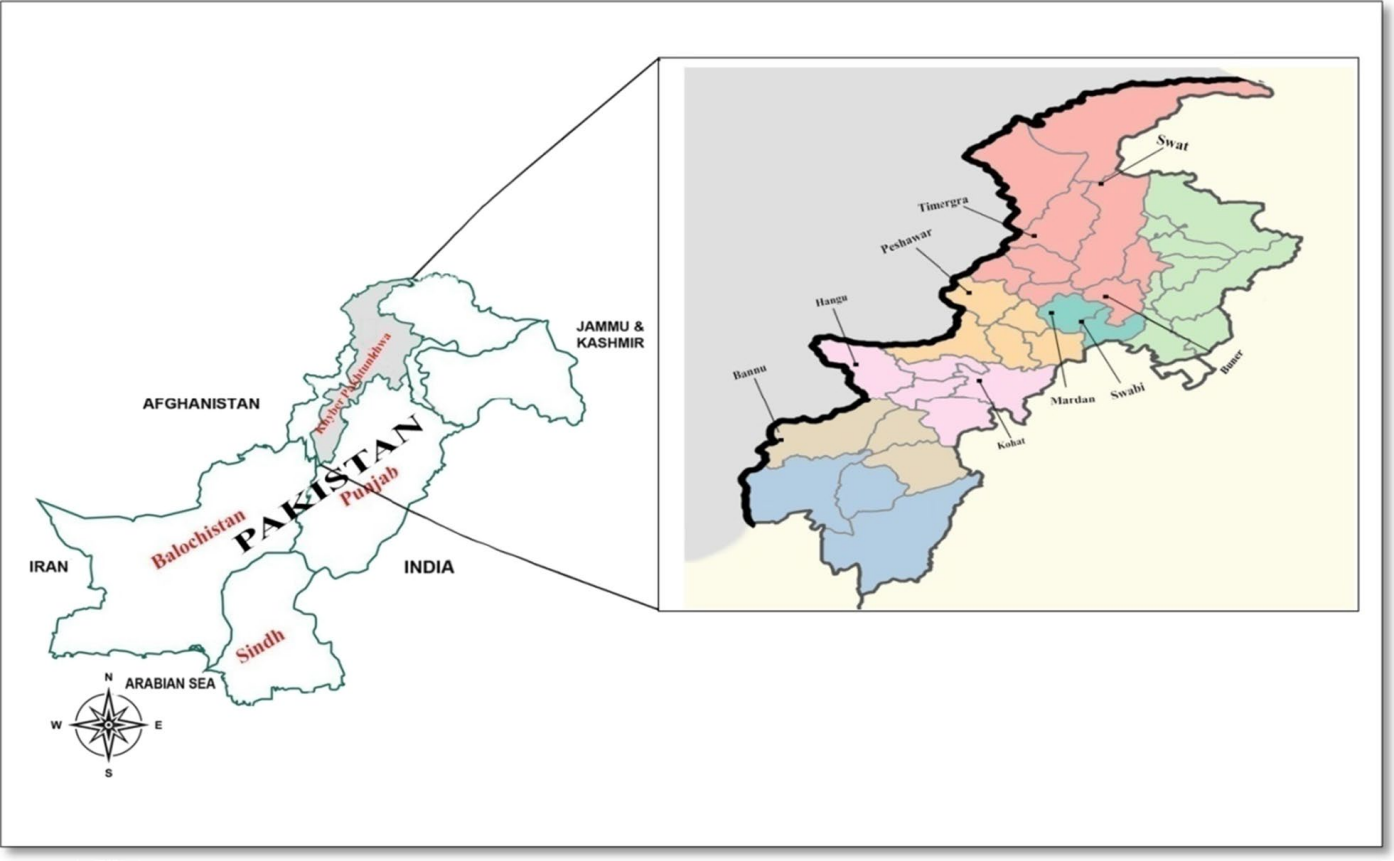
Map of Khyber Pakhtunkhwa (KP), Pakistan. The different districts from where the samples are collected have been marked

The domain-I of AMA-1 exhibits high level genetic polymorphism and this region appears to be a major target of anti-AMA-1 protective antibodies [19–22]. It is therefore noteworthy to monitor genetic variations of the vaccine candidate antigens among global malaria pathogenic isolates circulating in endemic areas, in order to design effective vaccine [23]. Several studies about antigenic variation of *Plasmodium vivax ama-1* (*pvama-1*) have been conducted in malaria endemic countries [24–28]. However, limited studies are reported about *pvama-1* genetic features from Pakistan. Particularly, no study till date is reported from remote malaria endemic regions of Khyber Pakhtunkhwa (KP) province of Pakistan. The current study was therefore designed to evaluate the genetic composition of *pvama-1* among *P. vivax* isolates collected from widespread KP regions of Pakistan (Fig. 1).

## Methods

### Study design, samples collection and DNA purification

The current study was approved from ethical review committee of Abdul Wali Khan University Mardan (AWKUM/Biochem/Dept/Commit/ECR/18). Blood samples were obtained from 100 consented patients tested positive for *P. vivax* using microscopy and rapid diagnostic test, while examined in different hospitals and private sector laboratories from Mardan, Swat, Buner, Hangu, Swabi, Kohat, Bannu, Timergara and Peshawar districts of KP province, Pakistan (Fig. 1). The region have an average annual rain fall of 384 mm during the two malaria seasons from March to May and from August to November. The mean temperature in the region ranges from 20 to 40 °C. The blood samples from the patients were collected prior to treatment, spotted on filters, air-dried, and kept in individual sealed plastic bags at ambient temperature until use. The genomic DNA was extracted from the spotted blood samples using a QIAmp blood kit (Qiagen, CA, USA) according to manufacturer’s instructions. The DNA samples were stored at − 20 °C.

### PCR amplification and DNA sequencing of *pvama-1* DI

A DNA fragment flanking the DI region of *pvama-1* was amplified by polymerase chain reaction (PCR) using the specific primers and amplification conditions as reported previously [21, 29]. The resulted PCR products were analyzed on 1.5% agarose gel, purified, and cloned into the T&A vector (Real Biotech Corporation, Banqiao City, Taiwan). Ligation mixture was transformed into *Escherichia coli* DH5α competent cells, and positive clones were selected by colony PCR. The nucleotide sequence of cloned insert was analyzed by automatic DNA sequencing with M13 forward and reverse primers (Genotech Inc., Daejoen, Korea). The raw data was filtered for quality assessment using DNASTAR Lasergene package.

### DNA polymorphism analyses

The DNA sequences data generated in the current study was analyzed in comparison with reference *pvama-1* sequence i.e. Sal-I (AF063138) and Genbank-deposited *pvama-1* sequences from China Myanmar Boarder (KX495505–KX495577), Iran (KF422636.1–KF422681.1), Korea (KM230319.1–KM230384.1), Myanmar (FJ157248.1–FJ157285.1), Papua New Guinea (PNG) (KC702402.1–KC702501.1), Sri Lanka (EF218679.1–EF218701.1), Venezuela (EU346015.1–EU346087.1), Thailand (FJ784891.1–FJ784990.1), and India (EU282774.1–EF025196.1). The comparative sequences analyses were performed using MEGA4 software suite [30] to identify and evaluate the polymorphic loci.

### Functional prediction of nsSNPs

The BepiPred-2.0 [31] server was used for prediction of Linear B-cell epitopes of *pvama-1* with a threshold score of **≥** 0.5. The higher BepiPred score predicts higher binding affinity of epitopes with immune receptors. The non-synonymous SNPs (nsSNPs) mapping within the top predicted epitopes of *pvama-1* was checked. The intrinsically unstructured regions (IURs) and RBC binding sites within the *pvama-1* have previously been characterized [25, 32]; and their annotation features were adopted in current study to check the nsSNPs mapping within these motifs of *pvama-1*. Additionally, the positive selection sites in B-cell epitopes were identified via the maximum likelihood method of Codeml [33] implemented in EasyCodeML [34].

### Statistical and population genetic analyses

The DnaSP v6.12 software package [35] was used to estimate parsimony informative sites, total number of mutations, pairwise nucleotide diversity (π), segregating sites (S), haplotypes (H) composition of the sequences, haplotypes diversity (Hd), recombination (R) between adjacent nucleotides per generation and the minimum number of recombination events (Rm). Additionally the linkage disequilibrium (LD) was estimated between the various polymorphic sites based on the R^2^ index via DnaSP [35]. The Tajima’s D, Fu and Li’s D* and F* indices were calculated via a sliding window method using DnaSP. The population genetics statistical analyses, including pairwise fixation index (*Fst*), analysis of molecular variance (AMOVA), haplotype frequencies, and nucleotide diversity based on Nei’s net distance (*DA*) were computed using Arlequin v3.5 [36]. The haplotype networking analysis and plot were generated using PopArt software [37].

## Results

### Genetic polymorphic features of KP *pvama-1*

The 416 bp sequences of *pvama-1*, flanking the DI domain were amplified from genomic DNA of 84 *P. vivax* positive samples. The sequences data spanning the 322–737 nucleotide positions of the reference *pvama-1* sequence i.e. Sal-I (AF063138). The analyses identified a large numbers of single nucleotide polymorphisms (SNPs) in KP samples. Among these, the 68 were nsSNPs, i.e. causing amino acid substitutions, including 53 dimorphic, 10 trimorphic, 3 tetramorphic, and 2 pentamorphic nsSNPs. The two pentamorphic amino acid changes observed were R112K/T/E/S and S228D/N/R/K. The ten trimorphic amino acid substitutions include the N132D/G, A141E/G, E145A/G, K190E/Q, T191K/P, A199T/V, S209G/C, P210S/L, P223L/S, and V233L/P. While the three tetramorphic amino acid changes are K120R/S/G, E189N/K/G, and E227V/K/G. These amino acid substitutions were observed at varied frequencies in the KP samples. Among the 68 nsSNPS, 59 have previously been reported in literature for *P. vivax* isolates from different geographical origin. However, the rest of 9 nsSNPs were found specific to KP samples set of this study. These nsSNPs were observed at low frequencies, i.e. 1.1 to 1.19%. Few nsSNPs, including K120R, N132D, L140I, A141E, K190E, E227V, and S228D were commonly observed in KP samples, as well as in some other continental *pvama-1* sequences with high frequency of 3.8–100% (Table 1). The KP *pvama-1* DI showed overall haplotype diversity (Hd) of 0.978 ± 0.008 (Additional file 1: Fig. S1). A total of 62 segregating sites (S) and 67 mutations were identified for the samples. The Fu and Li’s D* and F* test and Tajima’s D were calculated to check the deviation from neutrality, and to identify whether natural selection have shaped the genetic composition of the *pvama-1* D1 region. The Fu and Li’s D* and F* tests results were found significantly negative for KP samples data (Table 2). The Tajima’s D value was negative, i.e. − 1.490, however not significant (*P* > 0.10). The difference between dN/dS ratio for *pvama-1* DI region was also found negative (− 0.05413 ± 0.02) in case of KP samples set.

**Table 1:** The nsSNPs identified in KP, Pakistan *P. vivax* samples in comparison to the reference *pvama-1* sequence SalI (AF063138)

1	^c^R112K/T/E/S	15	L140I^a^	29	^c^V170A	43	E201G	57	^c^N226D
2	^c^P113S	16	A141E^a^	30	^c^M171T	44	^c^M203T	58	^c^E227V^a^
3	^c^G117R^b^	17	N142D	31	^c^A172T	45	^c^G204D	59	^c^S228D^a^
4	^c^D118N	18	^c^K144T	32	V184A	46	^c^R206G	60	N231D^b^
5	^c^Q119H	19	^c^E145A/G	33	K188N	47	^c^S209G^b^	61	V233L/P
6	^c^K120R^a^	20	^c^K148Q	34	E189N/K/G	48	^c^P210S/L^a^	62	Y234H^b^
7	^c^F126S	21	^c^D149N	35	K190E^a^	49	^c^A212V^b^	63	L235S
8	^c^N130K	22	^c^M153T	36	T191K/P	50	^c^N214S	64	N238D^b^
9	^c^A131T	23	I159T	37	C192R	51	^c^R215T	65	^c^R240C
10	^c^N132D/G^a^	24	A160T	38	H193Y	52	^c^V218L	66	^c^N241D
11	^c^D133N	25	L161V	39	M194V	53	^c^F221L	67	^c^D242E
12	^c^H134R	26	C162R^b^	40	Y196H^b^	54	^c^K222N	68	^c^W243R
13	^c^S136T	27	A166P	41	S198P	55	^c^P223L^b^		
14	T139A	28	A167P	42	A199T/V	56	^c^K225E		

^a^The common nsSNPs identified in KP and other *P. vivax* samples deposited in Genbank, NCBI

^b^Novel nsSNPs identified only in case of newly sequenced KP, Pakistan samples analyzed in the current study

^c^nsSNPs mapped within the predicted B-cell epitopes

**Table 2:** The neutrality test and genetic polymorphisms estimation for *pvama-1* domain-1 DNA sequences of KP, Pakistan and global samples

Countries	n	S	S(si)	Parsimony informative sites	η	K	H	Hd ± SD	π ± SD	Tajima’s D	D*(F&L)	F*(F&L)	References
Pakistan	84	62	41	21	67	7.335	57	0.978 ± 0.008	0.01763 ± 0.00084	− 1.490 *P* > 0.10	− 5.167 *P* < 0.02	− 4.418 *P* < 0.02	Current study
China-Myanmar boarder	73	22	3	19	24	6.249	25	0.914 ± 0.021	0.01502 ± 0.00077	0.81996 *P* > 0.10	0.00217 *P* > 0.10	0.36221 *P* > 0.10	Zhu et al. [25]
Iran	80	19	4	17	23	7.101	30	0.975 ± 0.010	0.01707 ± 0.00072	1.17202 *P* > 0.10	0.43585 *P* > 0.10	0.81792 *P* > 0.10	Esmaeili Rastaghi et al. [36]
Korea	66	23	4	19	23	4.205	15	0.782 ± 0.047	0.01011 ± 0.00111	− 0.40425 *P* > 0.10	0.32761 *P* > 0.10	0.07398 *P* > 0.10	Kang et al. [26]
Myanmar	38	45	23	22	47	8.580	37	0.999 ± 0.006	0.02063 ± 0.00091	− 0.83341 *P* > 0.10	− 1.88701 *P* > 0.10	− 1.80822 *P* > 0.10	Moon et al. [21]
PNG	100	21	2	19	22	6.10667	28	0.941 ± 0.009	0.01468 ± 0.00057	1.28215 *P* > 0.10	0.95445 *P* > 0.10	1.28946 *P* > 0.10	Arnott et al. [22]
Sri Lanka	23	15	3	12	15	0.01024	9	0.858 ± 0.047	0.01024 ± 0.00158	0.17272 *P* > 0.10	0.44859 *P* > 0.10	0.42662 *P* > 0.10	Gunasekera et al. [24]
Venezuela	73	12	0	12	13	4.718	12	0.847 ± 0.019	0.01134 ± 0.00044	2.15646 *P* < 0.05	1.43284 *P* < 0.05	2.06299 *P* < 0.02	Ord et al. [39]
Thailand	100	18	1	17	23	6.6000	34	0.919 ± 0.015	0.01587 ± 0.00057	1.43285 *P* > 0.10	0.59998 *P* > 0.10	1.09630 *P* > 0.10	Putaporntip et al. [38]
India	59	23	4	19	26	7.10286	41	0.980 ± 0.008	0.01707 ± 0.000	0.86316 *P* > 0.10	0.21989 *P* > 0.10	0.53811 *P* > 0.10	Thakur et al. [37]

n: number of samples; S: number of polymorphic sites (Segregating sites); K: average number of pairwise nucleotide differences; *H*: haplotype; Hd: haplotype diversity; π: observed average pairwise nucleotide diversity; D* (F&L): Fu and Li’s D* value; F* (F&L): Fu and Li’s F* value

P value ≤ 0.05 is considered significant

### Haplotype networking analysis

**Fig. 2:**
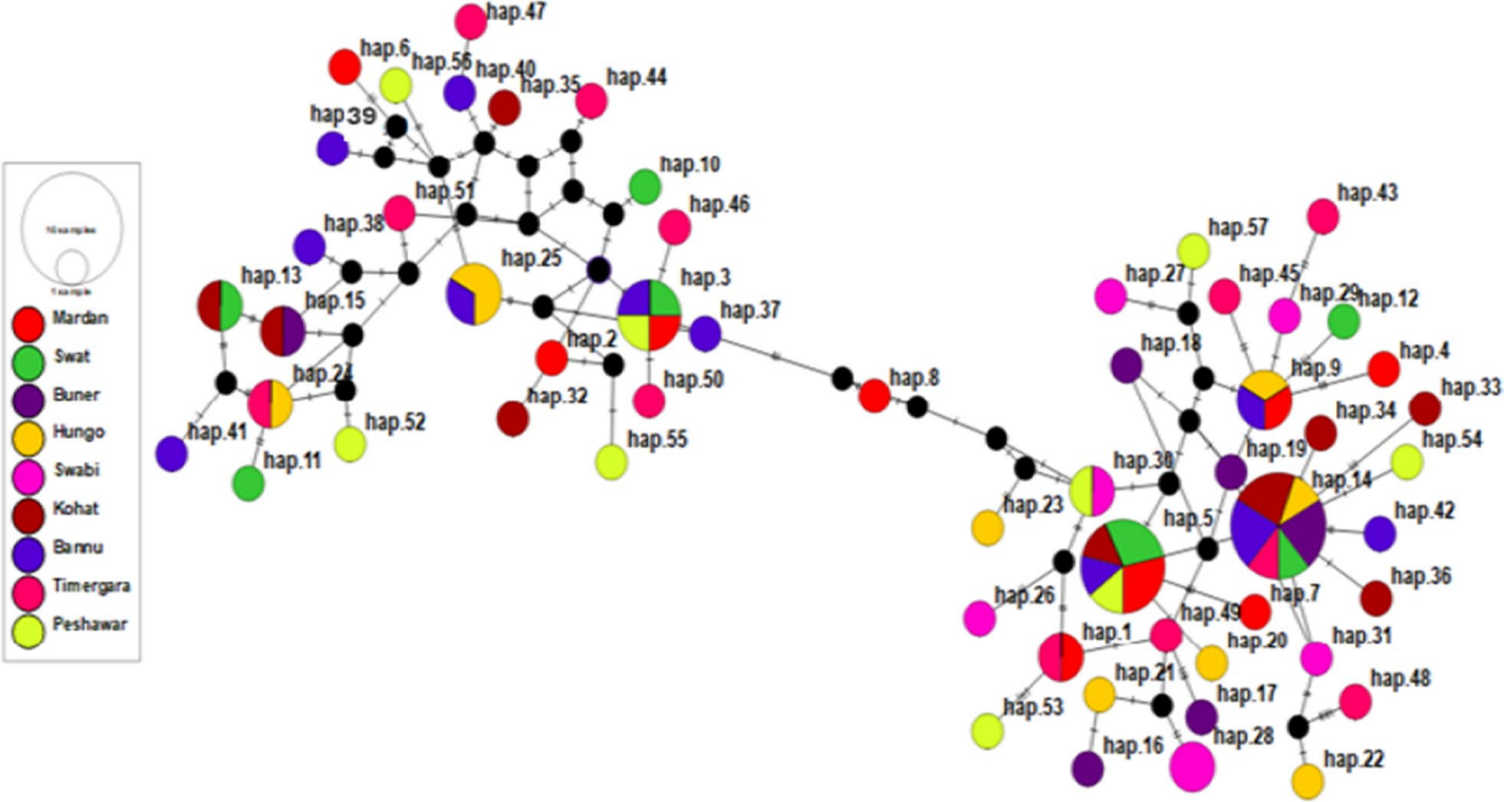
The Network plot of KP, Pakistan samples based on *pvama-1* DI sequences. The circle represents each haplotype, and the size of each circle is proportional to the number of samples holding that specific haplotype. The lines connecting the haplotypes reflect the distance of relatedness between haplotypes. The colors indicate the samples groups collected from different districts of KP, Pakistan

Total 57 haplotypes were identified for the 84 KP samples sequences of *pvama-1* DI (Additional file 2: Table S1). The H-14 haplotype was identified with high frequency and shared among samples collected from six different KP districts including, Kohat, Hungo, Buner, Swat, Timergara and Bannu. The H-5 haplotype was identified as second predominant haplotype shared among samples collected from five different KP districts (i.e. Mardan, Swat, Hungo, Bannu and Kohat). The H-3 haplotype was also identified with highest frequency among samples collected from Swat, Mardan, Peshawar and Bannu districts. The pairwise AMOVA inferred genetic distances among haplotypes. The H-53, i.e. predominant in Peshawar samples, was identified as distinct and showed significant genetic differentiation against the H-6 and H-55. The H-6 and H-55 were identified with high frequency in samples collected from Mardan and Peshawar regions respectively. The majorly shared haplotypes of KP samples collected from different districts appeared on share nodes of network plot, however, some haplotypes from Timergara, Peshawar, Kohat and Hungo samples occupied distinct nodes in the network plot, inferring their distinctive features (Fig. 2). The size of each node in haplotype network plot indicates the frequency of a particular haplotype. The length of the line between nodes is proportion to the number of nucleotide substitutions composing the haplotypes.

### Functional impact assessment of nsSNPs

The functional impact of the nsSNPs (Table 1) was assessed with respect to amino acids substitution in the unstructured/disordered regions, i.e. IURs, RBC binding region and B-cell epitopes of *pvama-1*. The IURs regions are considering important in vaccine designing and diagnosis. The analysis unveiled that two SNPs i.e., M171T, V172T mapped within the IURs motifs of pvama-1 DI domain. Four SNPs i.e. R240C, N241D, D242E and W243R are mapped within the RBC binding sites of *pvama-1*. Likewise, 40 nsSNPs, including four novel nsSNPs, i.e. found in newly sequenced KP samples, were mapped within the top predicted B-cell epitopes of *pvama-1* (Table 1). The B-cell epitopes were additionally examined for positive selection using the four site-specific models implemented in Easy CodeML [33], i.e. M0—one-ration vs. M3—discrete, M1a—nearly neutral vs. M2a—positive selection, M7 (β) vs. M8—β & ω > 1, and M8a—β & ω = 1 vs. M8—β & ω = 1. The likelihood ratio test (LRTs) was implemented to identify the site-specific positive selection with > 99% posterior probability. Total 15 residues were predicted underlined positive selection with P ≤ 0.05 in the *pvama-1* D1 domain region. These include the R112K, K120R, N130K, A131A, N132D, L140L, A141E, E145A, E189E, K190K, H193Y, P210P, E227E, S228S, and V233V. Among these, the nine SNPs, i.e. the R112K, K120R, N130K, A131A, N132D, E145A, P210P, E227E, and S228S are mapped within the B-cell epitopes.

### Recombination and linkage disequilibrium (LD) analyses

The KP samples sequences along with global sequences showed decline of LD index R^2^ with the increase of nucleotide distance which speculate high meiotic recombination events across the *pvama-1* region. The R value for KP samples were observed higher compare to China-Myanmar boarder, Korean, and Sri Lankan samples, while lower than the Myanmar samples sequences previously deposited in Genbank [21, 22, 24–26, 38–41]. The higher R value for isolates sequences from different regions depicts the opportunity of high multiclonal infections, cross fertilization and recombination [32]. The higher values of recombination and rapid LD decay as observed in case of some geographical samples speculating the recombination as a possible factor to provoke genetic diversity (Table 3). However, in case of KP samples slow LD decay across the nucleotide distance in *pvama-1* DI region was observed that inferring low nucleotide diversity (Additional file 3: Fig. S2).

**Table 3:** Recombination events estimation in *pvama-1* (Domain-1) region in KP, Pakistan and global *P. vivax* samples

Country/region	R^a^	R^b^	Rm
Pakistan	0.0892	37	5
China Myanmar	0.0506	21	5
Iran	0.0846	35.1	6
Korea	0.0022	0.9	2
Myanmar	0.1940	8.6	6
PNG	0.0513	21.3	5
Sri Lanka	0.0154	6.4	4
Venezuela	0.0381	15.8	4
Thailand	0.0879	36.5	5
India	0.1210	50.2	6

R^a^ recombinant parameter between adjacent sites, R^b^ recombinant parameter for the whole region, Rm minimum number of recombinant events

### Nucleotide diversity across *pvama-1* in context of global isolates

**Fig. 3:**
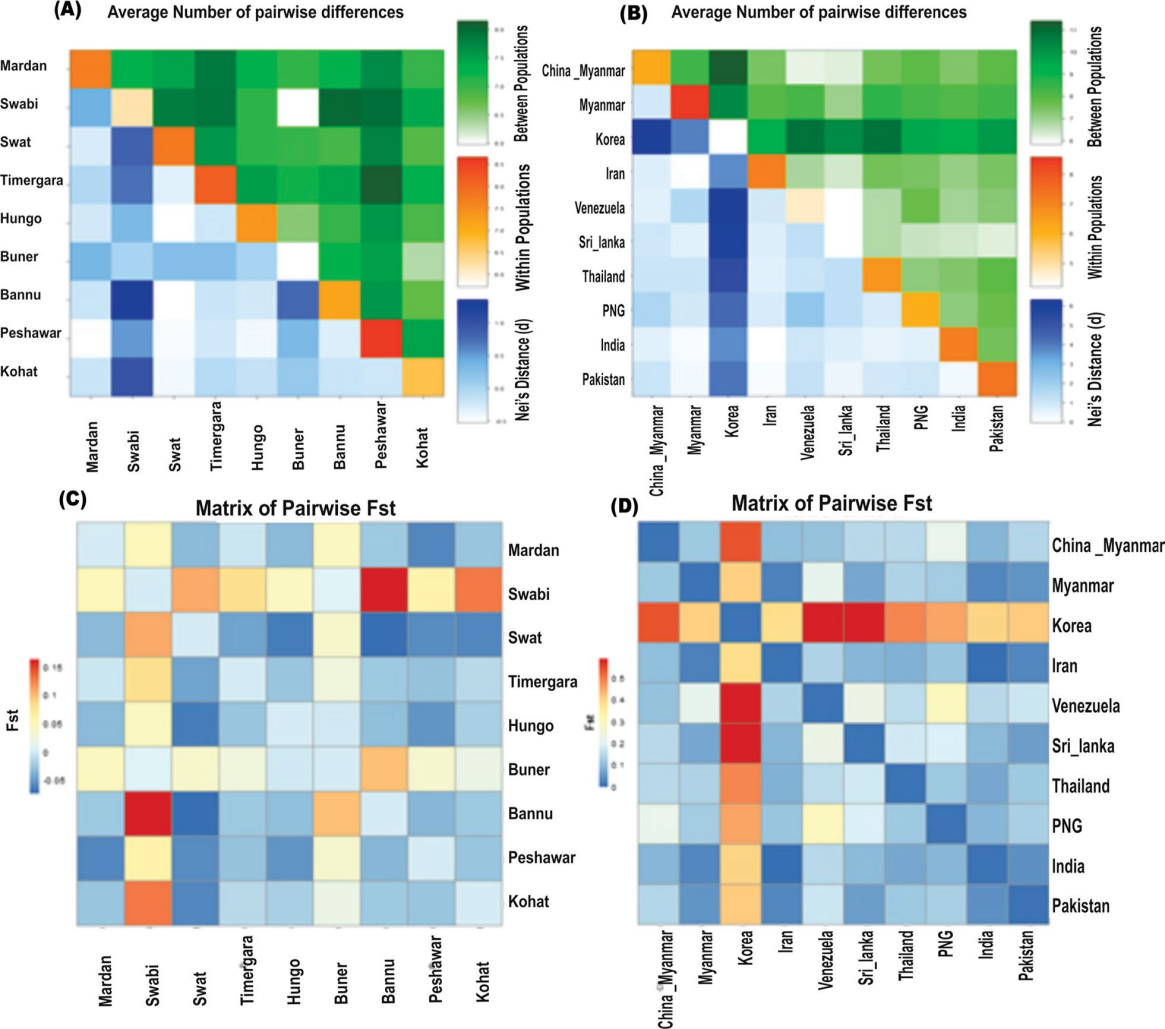
Pairwise population genetics analyses. **A** The graph represents the average number of pairwise differences (πxy), between the KP, Pakistan *P. vivax* samples population groups collected from different districts (Green above diagonal); within-population group πxx (orange diagonal) and the net number of nucleotide differences between population’s groups (Nei distance DA) (blue below diagonal) based on *pvama*-*1* DI gene variants. **B** The graph represents the average number of pairwise differences (πxy) between KP, Pakistani samples in context of global samples (Green above diagonal); within-population group πxx (orange diagonal) and the net number of nucleotide differences between population’s groups (Nei distance DA) (blue below diagonal) based on *pvama-1* DI variants. **C** Heat-map plot of pairwise *Fst* based on *pvama-1* gene sequences of KP, Pakistan samples groups collected from different districts. **D** Heat map of pairwise *Fst* distinction between KP, Pakistan *P. vivax* samples groups based on *pvama-1* gene sequences in context of worldwide sequences deposited in Genbank, NCBI.

The sequences of KP isolates (*n =* 84) were compared to the global *pvama-1* sequences deposited in Genbank. The values of K and π observed for KP sequences were more or less similar to previously reported sequences from Iran and India, however different from the rest of global sequences (Table 2). The fixation index *Fst* statistic was used to assess the genetic differentiation across *pvama-1* DI region among KP samples collected from different districts as well as in context of global samples. The pairwise analysis inferred genetic distinction of samples collected from Swabi district compare to rest of the KP regions. The top *Fst* differentiation was detected between the Bannu and Swabi isolates (*Fst* = 0.16258, *P*-value = 0.00977), followed by Swabi and Kohat (*Fst* = 0.12932; *P*-value = 0.04199) samples. The lowest *Fst* was estimated between Swat and Bannu samples (*Fst* = − 0.07427, *P*-value = 0.96973), followed by Swat and Hungo samples (*Fst*= − 0.06635, *P*-value = 0.89551) (Fig. 3A). The negative *Fst* is consider zero, depicting no genetic distinction between the population groups. The highest pairwise net number of nucleotide variation (*DA*) and mean pairwise differences (π_xy_) was observed between Bannu and Swabi samples, i.e. congruent to the *Fst* result (Fig. 3B). In context of global samples, marked genetic distinction inferred for KP samples compare to India, Iran, Thailand, Sri-Lanka, Korea, Venezuela, Myanmar, PNG, and China-Myanmar. Highest pairwise genetic differentiation was observed between KP and Korean samples (*Fst* = 0.40915). The Korean samples showed significant genetic distinction in pairwise comparison to rest of the global samples as well. Meanwhile, least genetic differentiation was observed among KP, Iranian, and Indian samples (Fig. 3C). The highest within population genetic differentiation (π) was found for Korean samples followed by South East Asian samples (Fig. 3D). Pearson correlation plot showed relationship among KP, Sri Lanka, Iran, India and Myanmar samples, congruent to pairwise *Fst* (Additional file 4: Fig. S3). The plot showed correlation among the populations in hierarchical order. However, the Korean samples showed high genetic distinction in term of *Fst* value, probably due to geographical separation (Additional file 5: Fig. S4).

AMOVA test determines the degree of regional variations and homogeneity within and among the isolates. We employed the AMOVA test to determination the genetic variation at single and multiple loci due to variation within a population group as well as between population groups. The AMOVA analysis depicted that genetic diversity in KP samples mainly arose due to within group differentiation i.e. 100.24%, instead of among groups genetic differentiation (− 0.24%). Likewise, higher variance component was noticed within the population group i.e. 3.67534 as compare to among populations (Table 4). Overall, the AMOVA test results inferring less genetic variation among KP isolates, collected from different wide-spread districts of KP region of Pakistan.

**Table 4:** AMOVA-based genetic differentiation analysis across the *pvama-1* (domain-1) in KP, Pakistan samples

Source of variation	d.f^a^	Sum of squares deviation (SS)	Estimates of variance components	Percentage of total variance contributed by each component	P-value
Among populations	8	28.742	− 0.00888 Va	− 0.24%	P = 0.50556
Within populations	75	275.650	3.67534 Vb	100.24%	P = 0.50556
Total	83	304.393	3.66646		

Local F*st* = − 0.00242

^a^d.f: degrees of freedom

## Discussion

The comprehensive knowledge about the antigenic variants in *Plasmodium* parasites is perquisite to design effective vaccine strategies workable in different endemic regions [42]. The current study aimed to analyze genetic composition of *pvama-1*, a leading malaria vaccine candidate antigen, among *P. vivax* isolates collected from different districts of KP, Pakistan.

The southern and northern regions of KP province of Pakistan are distinct with respect to geographical and environmental perspectives. However, limited genetic diversity of *P. vivax* across *pvama-1* DI domain was identified in the current study, suggesting no significant genetic heterogeneity among the *P. vivax* isolates from southern to northern KP regions. The low genetic diversity across the DI domain of *pvama-1* in KP regions might arose due to low endemicity of *Plasmodium* genotypes, as the low endemic region is generally characterized with limited parasitic genetic diversity [43, 44]. The low transmission and endemicity of *P. vivax* in the KP, Pakistan might have been provoked due to active malaria control programs in these regions from last several years. The pairwise genetic analyses unveiled more or less homogeneous genetic composition of KP samples to South/Central Asian samples from India and Iran regions. This might be due to close geographical contacts among these countries. The excess of segregating sites and significant negative Fu and Li’s D* and F* tests results indicate an excess of rare alleles in KP samples that might result from selective sweep. Additionally these results indicate excess of singleton mutations emerging from rapid population growth and directional selection. More or less similar data pattern was observed for Myanmar isolates [21]. The directional selection lead towards fixing beneficial alleles in the population and causes reduce genetic diversity [45]. However, the negative value (− 0.05413 ± 0.02) of dN/dS ratio for KP isolates data predicted the scenario of purifying and negative selection [37, 38].

The analyses of KP samples in context of global samples inferred unique genetic features and 9 KP samples-specific nsSNPs were identified in the newly sequenced samples. The genetic polymorphisms identified in the current study were further analyzed with respect to their possible functional consequences in the predicted RBC binding sites, B-cell epitopes, and IURs regions of *pvama-1.* Several nsSNPs were found to be located at the predicted RBC-binding sites, B-cell epitopes and IURs region of *pvama-1*. The nsSNPs mapped at the B-cell predicted epitopic motifs indicating a high degree of positive selection across the B-cell epitopes region of *pvama-1*. Likewise, several *pvama-1* SNPs were detected in *pvama-1* IURs region. The IURs play an important role in molecular recognition, assembling and protein modification [46]. The *pvama-1* IURs are indispensable for attachment and invasion of the parasite into RBCs [47]. The protein structure affected by amino acid changes due to these nsSNPs may affect the physicochemical perspectives of the *P. vivax* AMA-1 protein that might help the parasite to escape from host protective immunity.

## Conclusion

The partial DNA sequencing and analyses of *pvama-1* DI domain unveiled limited genetic diversity of *pvama-1* across the KP regions. This somehow suggested that *pvama-1* based vaccine against *P. vivax* might be promising to effectively combat and contribute in malaria eradication throughout the KP province of Pakistan.

## Supplementary Information

**Additional file 1: Fig S1.** Frequency of different haplotypes detected in *pvama-1* DI of *P. vivax* samples collected from different districts of KP, Pakistan

**Additional file 2: Table S1.** The haplotypes identified in *pvama-1* DI region of KP, Pakistan *P. vivax* isolates.

**Additional file 3: Fig S2.** Linkage disequilibrium (LD) pattern of *pvama-1* DI sequences of *P. vivax* isolates from KP, Pakistan. The LD index (R^2^) (Y-axis) plotted against nucleotide distance (X-axis) using a two tailed Fisher’s exact test.

**Additional file 4: Fig S3.** Pearson correlation plot of KP and global *pvama-1* samples based on pairwise *Fst* values. The plot shows clustering and correlation between the groups in hierarchical order. The dark brackets and large sizes depict the minimum genetic distinction and high correlation.

**Additional file 5: Fig S4.** The principle component analysis (PCA) of *pvama-1* DI sequences. Different colors depict different populations groups. This include KP Pakistan (as a single group) and worldwide samples.

## Data Availability

All the relevant data is provided in Additional figures and tables. The entire DNA sequencing data generated during current study is available from Genbank, NCBI (https://www.ncbi.nlm.nih.gov/genbank/) under the accession numbers; ON238020**–**ON238103.

## Abbreviations

Pv: *Plasmodium vivax*
SNPs: Single nucleotide polymorphism
AMA: Apical membrane antigens
RBC: Red Blood Cells
DI: Domain I
PCA: Principal component analysis
LD: Linkage disequilibrium
PCR: Polymerase chain reaction
KP: Khyber Pakhtunkhwa
