# Supplementary material for: Population genetic analyses inferred a limited genetic diversity across the pvama-1 DI domain among Plasmodium vivax isolates from Khyber Pakhtunkhwa regions of Pakistan
Source: BMC Infect Dis. 2022 Oct 30;22:807. doi: 10.1186/s12879-022-07798-1 (PMC9620592; doi:10.1186/s12879-022-07798-1)
Supplement: Supplementary file 1 — Additional file 1: Fig S1. Frequency of different haplotypes detected in pvama-1 DI of P. vivax samples collected from different districts of KP, Pakistan [file 12879_2022_7798_MOESM1_ESM.docx]

**
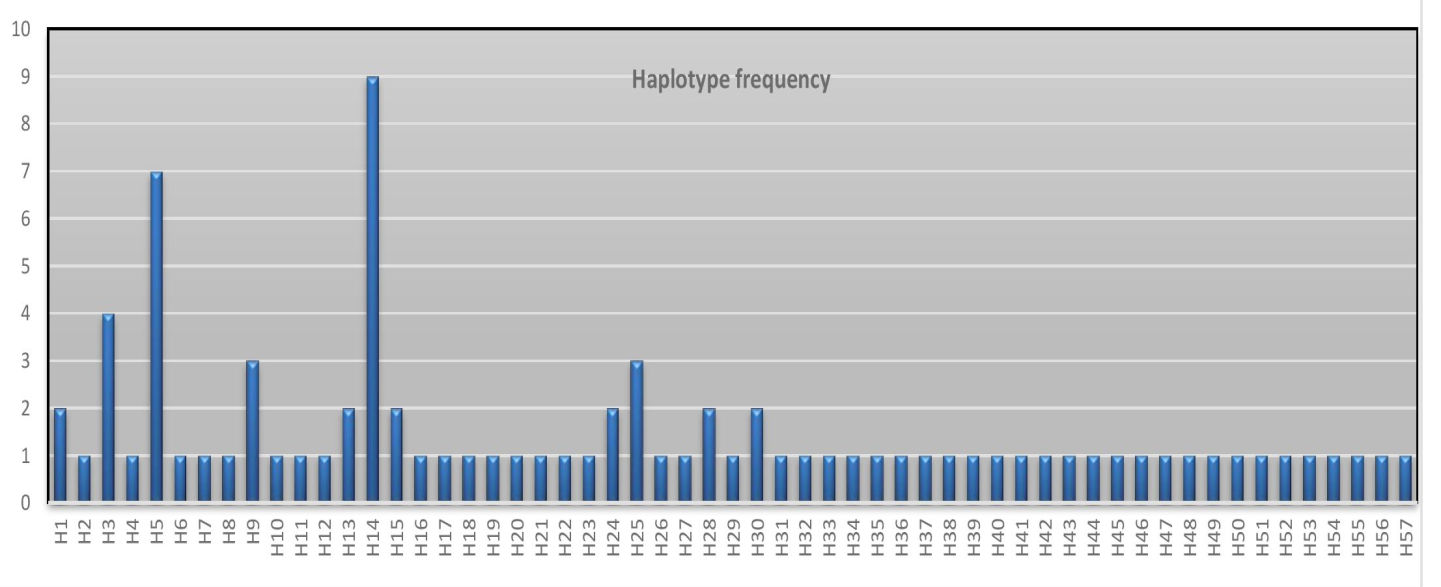
**

**Figure S1:** Frequency of different haplotypes detected in *pvama-1* DI of *P. vivax* samples collected from different districts of KP, Pakistan
