# Supplementary material for: Population genetic analyses inferred a limited genetic diversity across the pvama-1 DI domain among Plasmodium vivax isolates from Khyber Pakhtunkhwa regions of Pakistan
Source: BMC Infect Dis. 2022 Oct 30;22:807. doi: 10.1186/s12879-022-07798-1 (PMC9620592; doi:10.1186/s12879-022-07798-1)
Supplement: Supplementary file 2 — Additional file 2: Table S1. The haplotypes identified in pvama-1 DI region of KP, Pakistan P. vivax isolates. [file 12879_2022_7798_MOESM2_ESM.docx]

**Table S1**: The haplotypes identified in *pvama-1* DI region of KP, Pakistan *P. vivax* isolates.

| Haplotype frequency |
| --- |
| Hap_1 2 AACAGAGGGGGTCTAACACGGTGGAAAATTTCGATAACTCCGTGCAGAAAGCACGTTTTATA |
| Hap_2 1 AACAGAGGTGATCACACACGGTGAATAACTTCGATAACTCCGTGCAGTAGATACGTTTTATA |
| Hap_3 4 AACAGAGGTGATCACACACGGTGGAAAACTTCGATAACTCCGTGCAGTAGATACGTTTTATA |
| Hap_4 1 CCCAGAGGTGGTCTCAAACGGTGGAAAACTTTGATAACTCCGTGCAGAAAGCACGTTTTATA |
| Hap_5 7 AACAGAGGTGATCACACACGGTGGAAAACTTCGATAATTCCGTGCAGAAAGCACGTTTTATA |
| Hap_6 1 CCCAGAGGTGGTCTCAAACGGTGAAAGACTTCAATAATTCCGTGCAGTAGATATGTTTTATA |
| Hap_7 1 AACACAGGTAATCACACACGGCGGAAAACTTCGATAATTCCGTGCAGAAAGCACGTTTTATA |
| Hap_8 1 AACAGAGGTGATCTCACACGGTGGAAAATTTCGATAATTCCGTGCAGTAAAGACGTTTTATA |
| Hap_9 3 AACAGAGGTGGTCTCAAACGGTGGAAAACTTCGATAACTCCGTGCAGAAAGCACGTTTTATA |
| Hap_10 1 AACAGAGGTGATCACACACGATGAAACACTTCGATAACTCCGTGCAGTAGATACGTTTTATA |
| Hap_11 1 AACAGAGGGGGTCTAACACGGTGAAACACTTCGATAACCCCGTGCAGTAGATACGTTCTATA |
| Hap_12 1 AACAGAGGAGGTCTCAAACGGTGAAAGACTTCGATAACTCCGTGCAGAAAGCACGTTTTATA |
| Hap_13 2 CCCAGAGGGGGTCTAACACGGTGAATAACTTCGATAACTCCGTGCAGTAGATACGTTTTATA |
| Hap_14 9 AACAGAGGTGATCACACACGGTGGAAAACTTCGATAACTCCGTGCAGAAAGCACGTTTTATA |
| Hap_15 2 AACAGAGGGGGTCTAACACGGTGAATAACTTCGATAACTCCGTGCAGTAGATACGTTTTATA |
| Hap_16 1 AACAGAGGGGGTCTAACACGGTGAATAACTTCGATAACTCCGTGCAGAAAGCACGTTTTGTA |
| Hap_17 1 AACAGAGGGGGTCTAACACGGTGGAAACCTTCGATAATTCCGTGCGAAAAGCACGTTTTATA |
| Hap_18 1 AACAGAGGTGATCTAACACGGTGGAAAACTTCGATAACTCCGTGCAGAAAGCACGTTTTATA |
| Hap_19 1 AACAGAGGTGGTCTCACACGGTGGAAAACTTCGATAACTCCGTGCAGAAAGCACGTTTTATA |
| Hap_20 1 AACAGAGGTGATCACACACGGTGGAAAACTTCGATAATTTCGTGCAGAAAGCACGTTTTATA |
| Hap_21 1 AACAGAGGGGGTCTAACACGGTGAATAACTTCGATAACTCCGTGCAGAAAGCACGTTTTATA |
| Hap_22 1 AACAGAGGTGGTCACACGCGGTGAATAACTTCGATAACTCCGTGCAGAAAGCACGTTTTATA |
| Hap_23 1 AACAGAGGTGATCTCACACGGTGGAAAATTTCGATAATTCCGTGCAGGAAGCACCCTTTATA |
| Hap_24 2 AACAGAGGGGGTCTAACACGGTGAAACACTTCGATAACTCCGTGCAGTAGATACGTTTTATA |
| Hap_25 3 AACAGAGGTGGTCACACACGGTGAAAGACTTCGATAATTCCGTGCAGTAGATACGTTTTATA |
| Hap_26 1 CCCAGAGGGGGTCTCACACGGTGGAAAATTTCGATAATTCCGTGCAGAAAGCACGTTTTATA |
| Hap_27 1 AACAGAAGTGATCTCAAACGGTGGAAAACTTCGATAACTCCGTGTAGAAAGCGCGTTTTATA |
| Hap_28 2 AACAGAGGGGGTCTAACACGGTGAAACACTTCGATAGCTCCGTGCAGAAAGCACGTTTTATA |
| Hap_29 1 AACAGAGGTGGTCTCAAACGGTGGAAAACTTCGATAATTCCGTGCAGAAAGCACGTTTTATA |
| Hap_30 2 AACAGAGGTGATCTCACACGGTGGAAAATTTCGATAATTCCGTGCAGAAAGCACGTTTTATA |
| Hap_31 1 AACAGAGGTGGTCACACACGGTGGAAAACTTCGATAACTCCGTGCAGAAAGCACGTTTTATA |
| Hap_32 1 AACAGAGGTGATCACACACGGTGAATAACTTCGATAACTCCGTGCAGTAGATACGTTTTACA |
| Hap_33 1 AACAGAAATGATCACACACGGTGGGAAACTTCGATAACTCCGTGCAGAAAGCACGTTTTATA |
| Hap_34 1 AACAGAGGTGATCACACACAGTGGAAAACTTCGATAACTCCGTGCAGAAAGCACGTTTTATA |
| Hap_35 1 AACAGAGGTGGTCTCAAACGGTGGAAAACTTCGATAACTCCGTGCAGTAGATACGTTTTATA |
| Hap_36 1 AACAGAGGTGATTACACACGGTGGAAAACTTCGATAACTCCGTGCAGAAAGCACGTTTTATA |
| Hap_37 1 AACAGAGGTGATCACACACGGTGGAAAACTTCGATAATTCCGTGCAGTAGATACGTTTTATA |
| Hap_38 1 AACAGAGGTGGTCTAACACGGTGAATAACTTCGGTAACTCCGTGCAGTAGATACGTTTTATA |
| Hap_39 1 GACAGAGGTGGTCTCAAACGGTGAAAGACTTCGACAATTCCGTGCAGTAGATACGTTTTATA |
| Hap_40 1 AACAGAGGTGGTCTCAAACGGTGAATAACTTCGATAACTCCGTGCAGTAGATACGTTTTATA |
| Hap_41 1 CCCAGAGGGGGTCTAGCACGGTGAAACACTTCGATAACTCCGTGCAGTAGATACGTTTTATA |
| Hap_42 1 AACAGAGGTGATCACACATGGTGGAAAACTTCGATAACTCCGTGCAGAAAGCACGTTTTATG |
| Hap_43 1 AACAGAGGTGGTCTCAAACGGTGGAAAATTTCGATAATTCTGTGCAGAAAGCACGTTTTATA |
| Hap_44 1 AACAGAAGTGATCTCAAACGGTGAAACACTTCGATAACTCCGTGCAGTAGATACGTTTTATA |
| Hap_45 1 AACAGAGGTGGTCTCAAACGGTGGAAAATTTCGATAACTCCGTGCAGAAAGCACGTTTCATA |
| Hap_46 1 AACAGAGGTGATCACACACGGTGGAAAACTTCGATAACTCCGCGCAGTAGATACGTTTTATA |
| Hap_47 1 AACAGAGGTGGTCTCAAACGGTGAATAACTTCGATAACTCCGTGCAGTTGATACGTTTTATA |
| Hap_48 1 AACGGAGGTGGTCACACACGGTTAATAACTTCGATAACTCCCTCCAGAAAGCACCTTTTATA |
| Hap_49 1 AACAGAGGGGGTCTAACACGGTGGAAAACTTCGATAACTCCGTGCAGAAAGCACGTTTTATA |
| Hap_50 1 AACAGGGGTGATCACACACGGTGGAAAACTTCGATAACTCCGTGCAGTAGATACGTTTTATA |
| Hap_51 1 AACAGAGGTGATCTAACACGGTGAAAAACTTCGATAACTCCGTGCAGTAGATACGTTTTATA |
| Hap_52 1 AACAGAGGGGGTCTAACACGGTGAAAGACTTCGATAATTCCGTGCAGTAGATACGTTTTATA |
| Hap_53 1 AACAGAAAGGGTCTAACACGGTGGAAAATCTCGATAACTCCGTGCAGAAAGCACGTCTTATA |
| Hap_54 1 AATAGAGGTGATCACACACGGTGGAAAACTTCGATAACTCCGTGCAGAAAGCACGTTTTATA |
| Hap_55 1 AACAGAGGTGAACACACACGGTGAATAACTTCGATAATTCCGTGCAGTAGATACGTTTTATA |
| Hap_56 1 AACAGAGGTAGTCTCAAACGGTGAAAGACTTCGATGATTCCGTGCAGTAGATACGTTTTATA |
| Hap_57 1 AACAGAAGTGATCTCAAACGGTGGAAAACTCCGATAACTCCGTGCAGAAAGCACGTTTTATA |
