# Supplementary material for: Population genetic analyses inferred a limited genetic diversity across the pvama-1 DI domain among Plasmodium vivax isolates from Khyber Pakhtunkhwa regions of Pakistan
Source: BMC Infect Dis. 2022 Oct 30;22:807. doi: 10.1186/s12879-022-07798-1 (PMC9620592; doi:10.1186/s12879-022-07798-1)
Supplement: Supplementary file 3 — Additional file 3: Fig S2. Linkage disequilibrium (LD) pattern of pvama-1 DI sequences of P. vivax isolates from KP, Pakistan. The LD index (R2) (Y-axis) plotted against nucleotide distance (X-axis) using a two tailed Fisher’s exact test. [file 12879_2022_7798_MOESM3_ESM.docx]

**
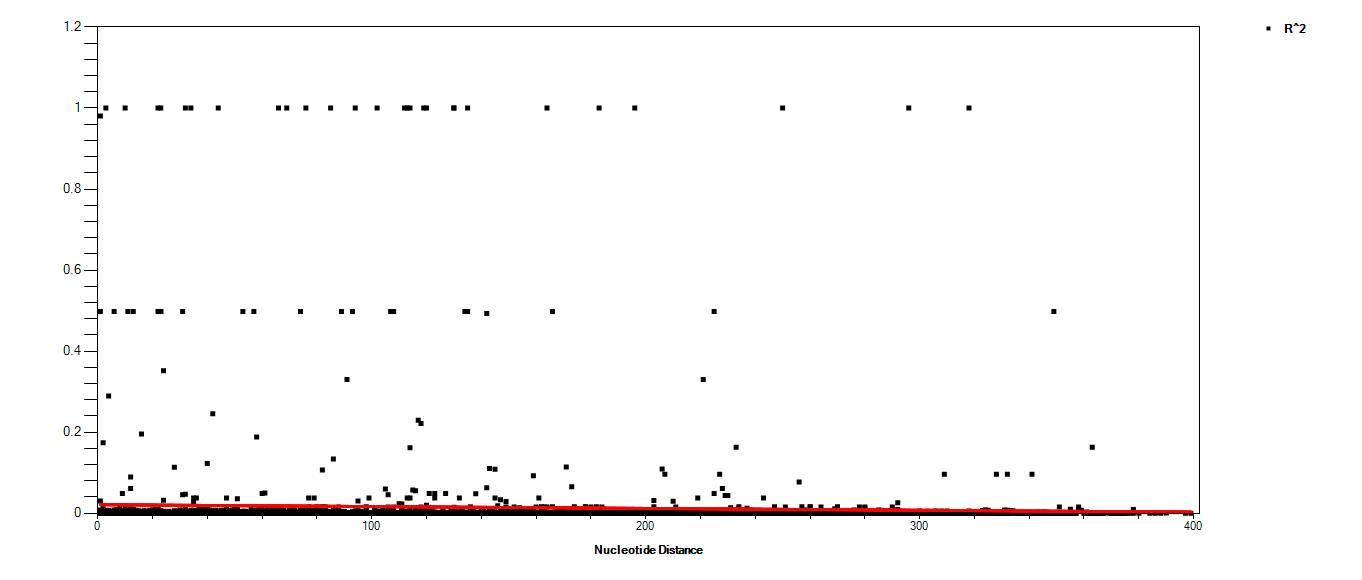
**

**Figure S2:** Linkage disequilibrium (LD) pattern of *pvama-1* DI sequences of *P. vivax* isolates from KP, Pakistan. The LD index (R^2^) (Y-axis) plotted against nucleotide distance (X-axis) using a two tailed Fisher’s exact test.
