# Supplementary material for: Population genetic analyses inferred a limited genetic diversity across the pvama-1 DI domain among Plasmodium vivax isolates from Khyber Pakhtunkhwa regions of Pakistan
Source: BMC Infect Dis. 2022 Oct 30;22:807. doi: 10.1186/s12879-022-07798-1 (PMC9620592; doi:10.1186/s12879-022-07798-1)
Supplement: Supplementary file 4 — Additional file 4: Fig S3. Pearson correlation plot of KP and global pvama-1 samples based on pairwise Fst values. The plot shows clustering and correlation between the groups in hierarchical order. The dark brackets and large sizes depict the minimum genetic distinction and high correlation. [file 12879_2022_7798_MOESM4_ESM.docx]

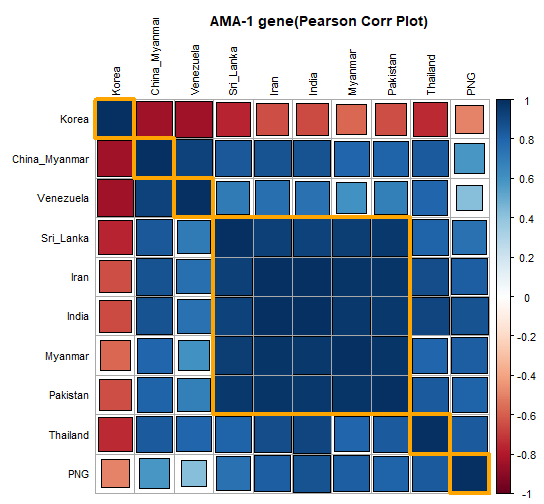


**Figure S3**: Pearson correlation plot of KP and global pvama-1 samples based on pairwise Fst values. The plot shows clustering and correlation between the groups in hierarchical order. The dark brackets and large sizes depict the minimum genetic distinction and high correlation.
