# Supplementary material for: Population genetic analyses inferred a limited genetic diversity across the pvama-1 DI domain among Plasmodium vivax isolates from Khyber Pakhtunkhwa regions of Pakistan
Source: BMC Infect Dis. 2022 Oct 30;22:807. doi: 10.1186/s12879-022-07798-1 (PMC9620592; doi:10.1186/s12879-022-07798-1)
Supplement: Supplementary file 5 — Additional file 5: Fig S4. The principle component analysis (PCA) of pvama-1 DI sequences. Different colors depict different populations groups. This include KP Pakistan (as a single group) and worldwide samples. [file 12879_2022_7798_MOESM5_ESM.docx]

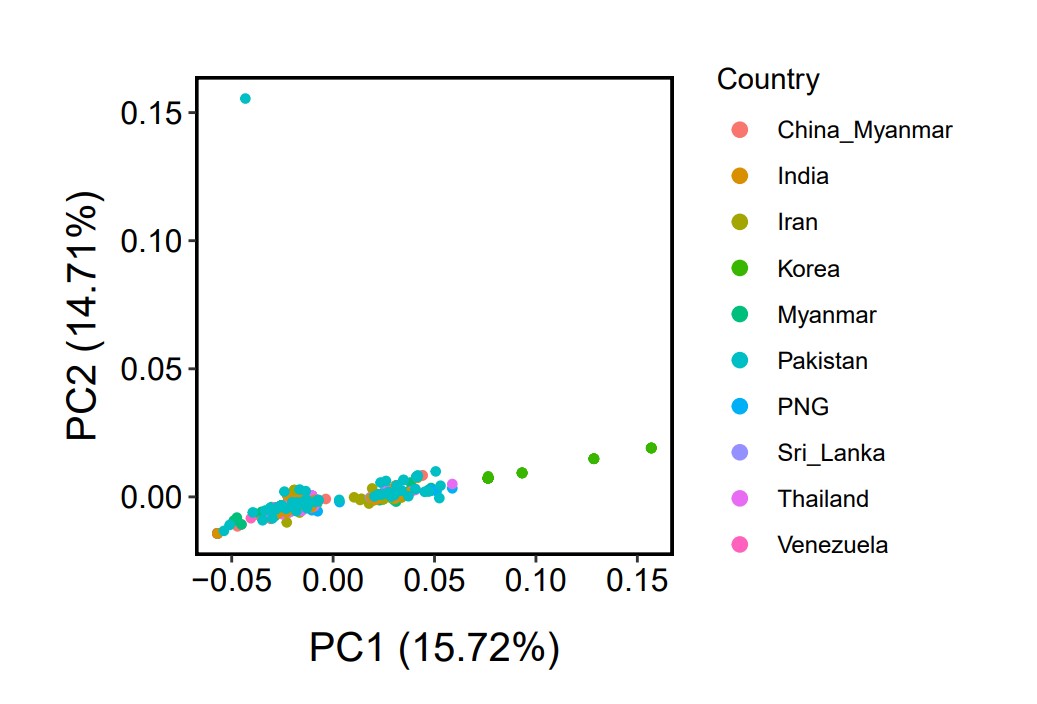


**Figure S4:** The principle component analysis (PCA) of *pvama-1* DI sequences. Different colors depict different populations groups. This include KP Pakistan (as a single group) and worldwide samples.
